# Unicentric Adrenal Castleman Disease Mimicking an Adrenal Tumour

**DOI:** 10.7759/cureus.110950

**Published:** 2026-06-16

**Authors:** Lin Aung Han, Yan Naung

**Affiliations:** 1 Urology, Southmead Hospital, Bristol, GBR; 2 Urology, Mandalay General Hospital, Mandalay, MMR

**Keywords:** adrenal lesion, hyaline vascular type, laparascopic adrenalectomy, lymphoproliferative disorder, open adrenalectomy, unicentric castleman’s disease

## Abstract

Castleman disease (CD) is an uncommon lymphoproliferative disorder of undetermined origin, also known as giant lymph node hyperplasia or angiofollicular lymph node hyperplasia. It is broadly classified into unicentric and multicentric forms, each exhibiting distinct clinical manifestations, pathological features, and prognostic implications. While the mediastinum remains the most frequently involved site, CD may occur in a variety of nodal and extranodal locations. Adrenal involvement is exceptionally uncommon and can pose a significant diagnostic challenge by mimicking primary adrenal neoplasms.

We report the case of a 24-year-old man who presented with nonspecific abdominal symptoms and was found to have a right adrenal mass. The patient underwent adrenalectomy, initially via a laparoscopic approach, which was subsequently converted to open surgery because of dense adhesions and proximity to major vascular structures. Histopathological examination established the diagnosis of unicentric hyaline vascular CD involving the adrenal region. To the best of our knowledge, this represents the first reported case of adrenal CD from Myanmar.

## Introduction

Castleman disease (CD) was first described by Benjamin Castleman at Massachusetts General Hospital in 1956. Despite increasing recognition, the aetiology and pathogenesis of CD remain incompletely understood [1]. The mediastinum is the most commonly affected site; however, extrathoracic involvement, including both nodal and extranodal presentations, has also been reported.

Castleman disease is a heterogeneous lymphoproliferative disorder that is classified according to the extent of disease involvement and its histopathological appearance. Localized disease, known as unicentric CD, is restricted to a solitary lymph node or nodal group, whereas multicentric CD involves multiple lymph node stations and is commonly associated with systemic inflammatory manifestations. The multicentric form may be linked to HHV-8 infection, polyneuropathy, organomegaly, endocrinopathy, monoclonal gammopathy, and skin changes (POEMS syndrome), or occur without an identifiable cause [2,3]. Microscopically, CD is recognized as hyaline vascular, plasma cell, or mixed type based on its predominant pathological features [4].

Adrenal involvement in CD is exceedingly rare and is often discovered incidentally during imaging. Radiological findings are usually nonspecific and may mimic more common adrenal neoplasms, making preoperative diagnosis challenging and often requiring histopathological confirmation. Surgical resection remains the treatment of choice for localized disease and is generally associated with an excellent prognosis [5].

We report an unusual case of unicentric CD presenting as a right adrenal mass, which was diagnosed following surgical excision. In addition, a review of previously reported cases is provided to enhance the understanding of the clinical presentation, diagnostic pitfalls, and management approaches for this rare adrenal manifestation of CD.

## Case presentation

A 24-year-old previously fit and healthy man presented to a private hospital with nonspecific abdominal pain associated with nausea and epigastric discomfort. His past medical and surgical histories were not significant. Physical examination was unremarkable, and standard laboratory investigations were within reference ranges.

Initial ultrasound examination of the abdomen demonstrated a retroperitoneal mass, likely arising from the right adrenal gland, measuring approximately 51 × 43 mm. Biochemical evaluation of adrenal function, including plasma free metanephrines and morning serum cortisol, was within normal limits, with no evidence of hormone excess to suggest pheochromocytoma or a cortisol-producing adenoma.

Subsequently, due to financial limitations, the patient underwent only a non-contrast-enhanced CT scan of the abdomen, which demonstrated a well-defined right adrenal mass measuring approximately 55 × 40 mm. Central calcification was noted, and part of the mass extended into the plane posterior to the right renal hilar vessels. CT showed no lymphadenopathy or additional tumours (Figures 1-3).

**Figure 1:**
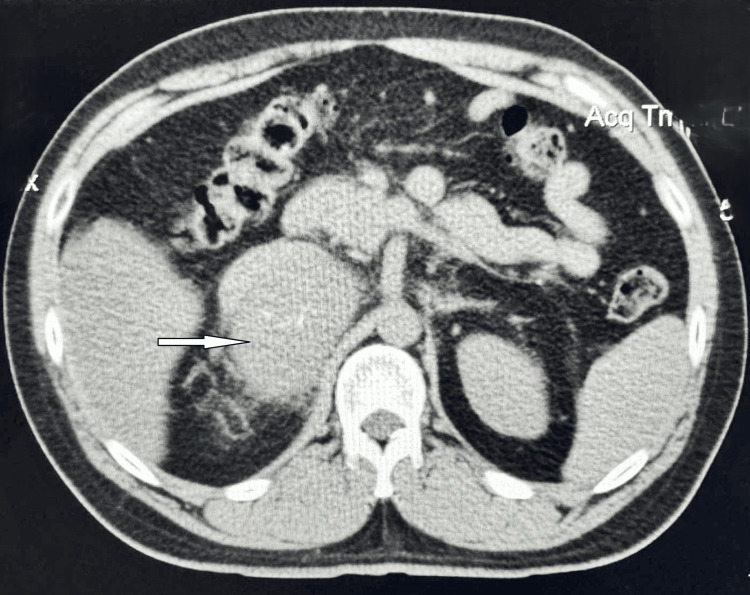
CT scan demonstrating a well-defined homogeneous soft-tissue-density mass (arrow) arising from the right adrenal gland, with maximum dimensions of approximately 55 × 40 mm.

**Figure 2:**
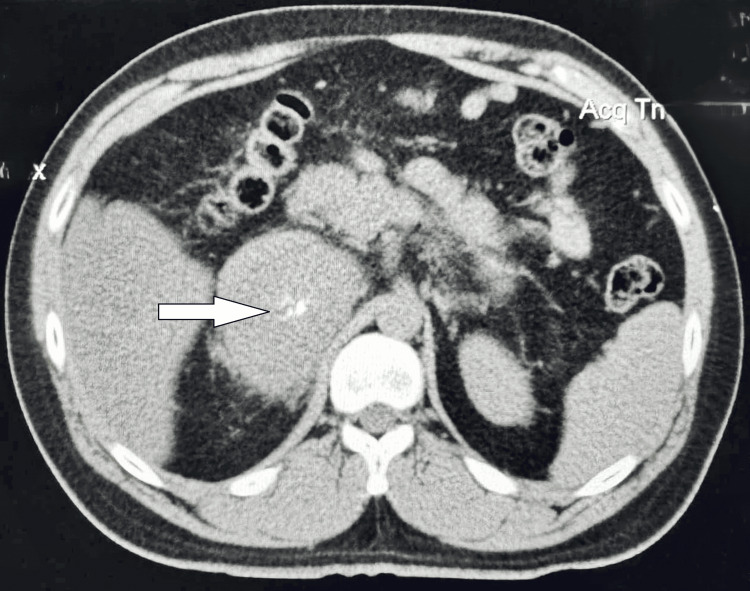
CT scan demonstrating a well-defined homogeneous soft-tissue-density mass with central calcification (arrow) in the right adrenal gland.

**Figure 3:**
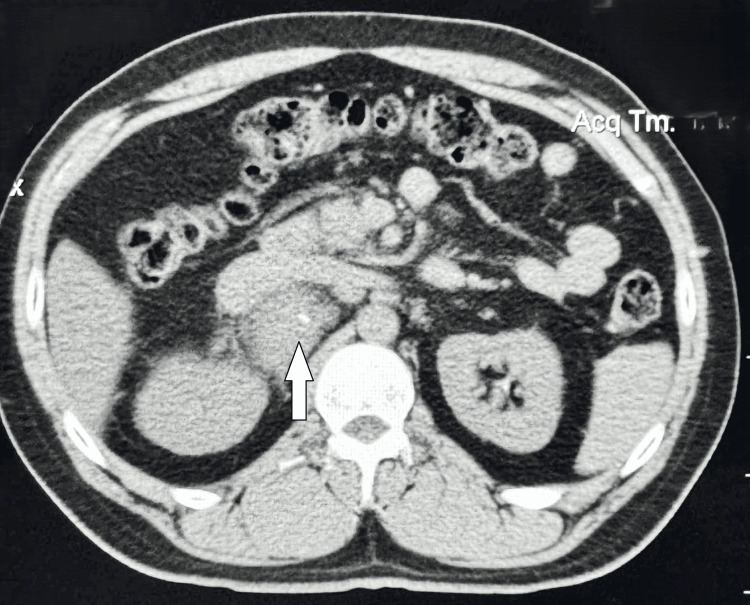
CT scan demonstrating a well-defined right adrenal mass (arrow) extending posterior to the right renal hilar vessels.

Following completion of the preoperative evaluation, the patient underwent laparoscopic resection of the right adrenal mass via a transperitoneal approach. Intraoperatively, the lesion was found to be densely adherent to the surrounding tissues and extended posterior to the right renal hilum. Due to the challenging dissection and close relationship of the mass to the renal hilar vessels, the procedure was converted to an open approach after initial laparoscopic attempts at mobilisation.

An open adrenalectomy was subsequently performed through a supra-12th-rib incision. Careful dissection allowed gradual separation of the mass from the renal hilar vessels and inferior vena cava. Estimated intraoperative blood loss was approximately 200 mL. The procedure was completed successfully without vascular injury or other intraoperative complications.

Gross pathological examination demonstrated a firm specimen measuring 60 × 50 × 38 mm and weighing 72.9 g. On sectioning, the tumour showed a solid, meaty cut surface.

Histologically, the tumour showed complete effacement of normal adrenal tissue by lymphoid stroma. It also revealed abnormal lymphoid follicles characterized by concentric mantle zone lymphocytes surrounding regressed germinal centres. Prominent hyalinised vascular proliferation and increased follicular dendritic cells were present, showing features typical of the hyaline vascular variant of CD. In the interfollicular areas, numerous proliferating vessels were noted. Foci of fibrosis and calcification were also seen. No malignant features were seen. The excision margins were free (Figures 4-6).

**Figure 4:**
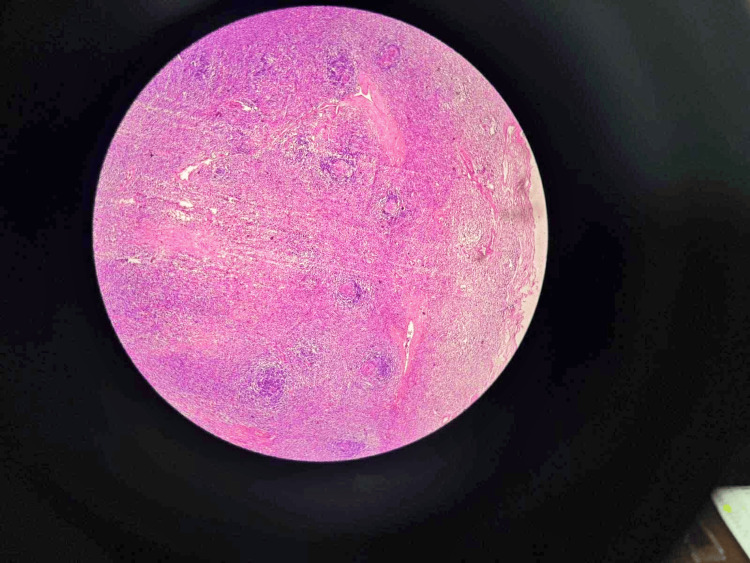
Histopathological examination showing lymphoid follicles with expanded mantle zones and a characteristic “onion-skin” arrangement surrounding atretic germinal centres, consistent with hyaline vascular-type Castleman disease (H&E stain, ×4 magnification).

**Figure 5:**
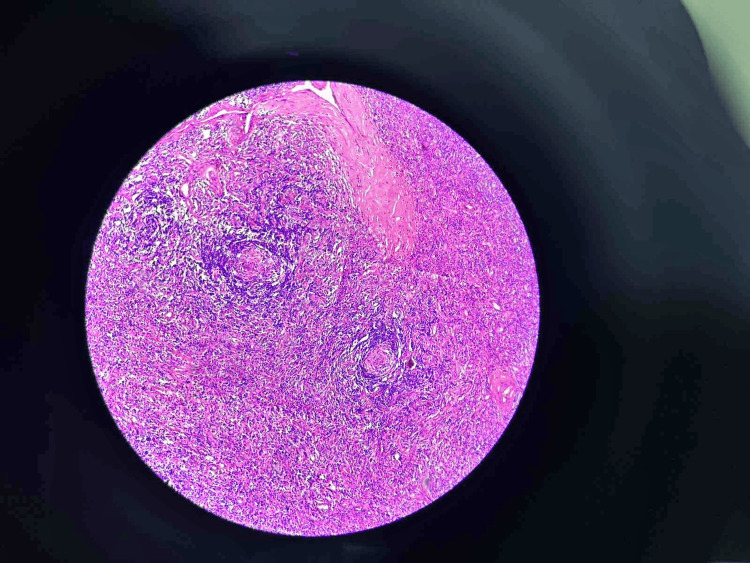
Histopathological section demonstrating regressed germinal centres with concentric layering of mantle zone lymphocytes (“onion-skinning”) and prominent hyalinised penetrating vessels, characteristic of hyaline vascular Castleman disease (H&E stain, ×10 magnification).

**Figure 6:**
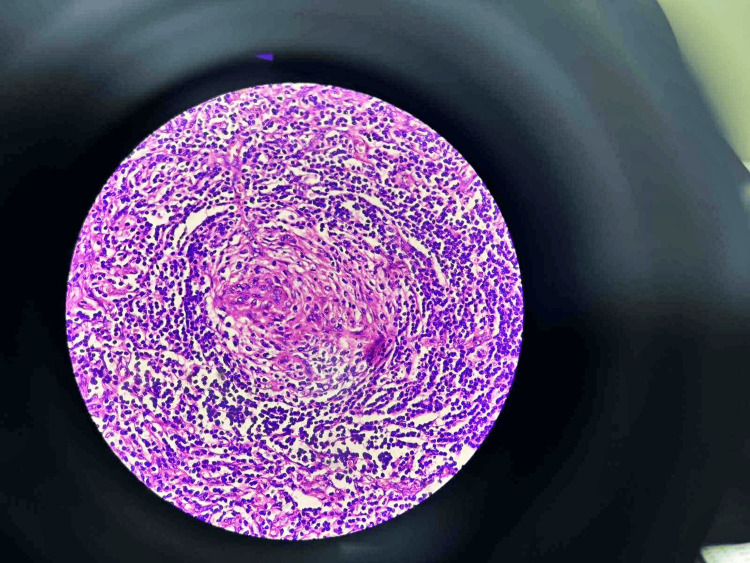
High-power histopathological view showing an atretic germinal centre penetrated by a hyalinised blood vessel (“lollipop” appearance) with concentric mantle zone layering, characteristic of hyaline vascular Castleman disease (H&E stain, 40x magnification).

The resected mass was ultimately identified as hyaline vascular-type CD of the adrenal gland on histopathological analysis. The postoperative course was uncomplicated, and no additional treatment was indicated. At 12 months of follow-up with abdominal ultrasonography, the patient remained asymptomatic, with no evidence of disease recurrence.

## Discussion

Castleman disease is a rare lymphoproliferative disorder that most commonly occurs in the mediastinum. Approximately 12% of cases arise within the abdomen, and adrenal involvement is exceptionally uncommon. To date, only a limited number of cases involving the adrenal gland have been reported in the literature [6].

The aetiopathogenesis of CD remains incompletely understood. Current evidence suggests that chronic inflammatory activity, immune dysregulation, and autoimmune processes may contribute to its development [4]. Published cases of adrenal CD have involved patients ranging in age from adolescence to late adulthood (16-65 years), with no apparent sex predominance. While abdominal pain is the most frequently reported symptom in unicentric adrenal disease, a substantial proportion of cases are identified incidentally during imaging performed for unrelated reasons [6].

CD can be classified according to both disease extent and histopathological features. Based on the extent of disease involvement, CD is classified into unicentric and multicentric forms. Based on microscopic features, CD is categorised into three histopathological subtypes: hyaline vascular, plasma cell, and mixed forms. Among these, the hyaline vascular subtype is by far the most frequently encountered, accounting for nearly 90% of unicentric cases [5]. It is characterised by abnormal lymphoid follicles, concentric mantle zone layering (“onion-skinning”), atretic germinal centres, and prominent vascular proliferation within the interfollicular regions. Patients with this subtype are usually asymptomatic. Nevertheless, when the lesion reaches a considerable size, symptoms related to local mass effect may occur, although constitutional manifestations are infrequently observed [7].

The plasma cell subtype is less common and is characterised histologically by sheets of mature plasma cells with relatively fewer vascular structures [8].

Clinically, CD is categorised into unicentric CD (UCD) and multicentric CD (MCD) [5]. UCD is the more common form and generally carries an excellent prognosis following complete surgical excision, whereas MCD is associated with systemic manifestations and a significantly poorer prognosis [9].

Imaging characteristics of hyaline vascular adrenal CD typically include a sharply defined, uniformly enhancing soft-tissue lesion on CT. Conversely, plasma cell-type disease tends to present as a larger mass with areas of necrosis, rim enhancement, and enlarged retroperitoneal lymph nodes, potentially mimicking a malignant adrenal tumour [10].

A characteristic radiological feature of hyaline vascular CD is the presence of a well-defined dominant mass, often associated with adjacent smaller nodules, showing intense enhancement following contrast administration as a result of its hypervascularity. Enhancement is usually homogeneous, although larger lesions may show heterogeneous enhancement secondary to central necrosis. Approximately 10% of hyaline vascular lesions contain internal calcifications [11]. In our case, central calcification and a relatively homogeneous appearance were observed; however, contrast-enhanced imaging was not performed.

Preoperative diagnosis of adrenal CD is particularly challenging because of its rarity and non-specific radiological features. It may mimic other hypervascular adrenal neoplasms on imaging studies. The differential diagnosis includes adrenocortical carcinoma and phaeochromocytoma. Adrenocortical carcinoma is typically large, often exceeding 6 cm, and commonly demonstrates heterogeneous enhancement due to necrosis. Invasion of adjacent vascular structures, particularly the inferior vena cava, may also be present [9]. Phaeochromocytoma demonstrates variable imaging characteristics. While small phaeochromocytomas are often homogeneous, larger lesions frequently exhibit mixed attenuation due to cystic change, calcification, or fatty degeneration. A typical imaging feature is intense contrast enhancement on CT, reflecting their hypervascular nature [12].

UCD involving a single anatomical site is generally benign, whereas multicentric disease progress to malignant lymphoma. Complete surgical excision remains the treatment of choice for unicentric hyaline vascular CD and is considered curative in most cases [6]. Recently published international consensus guidelines for UCD also recommend complete surgical resection as first-line management [13].

Although minimally invasive approaches, including laparoscopic and robotic adrenalectomy, may reduce postoperative pain, shorten hospital stay, and facilitate faster recovery, conversion to open surgery may occasionally be necessary in technically challenging cases. In our patient, the lesion extended posterior to the renal hilum and was densely adherent to the surrounding structures, necessitating conversion to an open procedure to ensure safe dissection and vascular control.

Recurrence of UCD following complete excision is uncommon, with reported recurrence rates below 5% [14]. Although regular postoperative surveillance with interval imaging is advised, there is currently no universally accepted follow-up strategy because of the limited number of reported cases.

MCD represents a more aggressive clinical entity and usually presents at an older age than unicentric disease. MCD carries a poorer prognosis and may exhibit malignant behaviour. Although the optimal treatment strategy remains uncertain, various modalities, including chemotherapy, corticosteroids, radiotherapy, and combination therapies, have been used with variable success. Despite therapeutic advances, mortality rates remain high, approaching 50%, highlighting the need for further research and improved treatment strategies for MCD [15].

## Conclusions

In conclusion, we present a rare case of hyaline vascular-type adrenal CD in a 24-year-old man who presented with non-specific abdominal symptoms and radiologically mimicked other adrenal neoplasms. Unicentric hyaline vascular CD masquerading as an adrenal tumour is an exceptionally uncommon clinical presentation. Because adrenal CD is exceedingly rare, establishing a preoperative diagnosis remains challenging, and definitive diagnosis is often achieved only after histopathological examination of the resected specimen. In patients with localised hyaline vascular disease, surgical management provides both diagnostic clarification and durable disease control. Outcomes are generally favourable following complete removal of the lesion, with recurrence being exceedingly uncommon. This case further expands the limited evidence on adrenal involvement in CD and underscores the need to include this rare condition among other potential causes of adrenal masses.
